# Development of musculoskeletal toxicity without clear benefit after administration of PG-116800, a matrix metalloproteinase inhibitor, to patients with knee osteoarthritis: a randomized, 12-month, double-blind, placebo-controlled study

**DOI:** 10.1186/ar2315

**Published:** 2007-10-24

**Authors:** Piotr Krzeski, Chris Buckland-Wright, Géza Bálint, Gary A Cline, Karen Stoner, Robert Lyon, John Beary, William S Aronstein, Tim D Spector

**Affiliations:** 1 Procter & Gamble Pharmaceuticals, Whitehall Lane, Egham TW20 9NW, Surrey, UK; 2 Applied Clinical Anatomy, King's College London School of Medicine, Hodgkin Building, London Bridge, London SE1 1UL, UK; 3 National Institute of Rheumatology and Physiotherapy, Frankel Leo u.38-40, 1024 Budapest, Hungary; 4 Procter & Gamble Pharmaceuticals, 8700 Mason-Montgomery Road, Mason, OH 45040-8006, USA; 5 Twin Research Unit, St Thomas' Hospital Campus, King's College London, Lambeth Palace Road, London SE1 7EH, UK

## Abstract

We performed a randomized, double-blind, placebo-controlled, multicenter, parallel-group, dose-response study of the efficacy and safety of the oral administration of PG-116800, a matrix metalloproteinase (MMP) inhibitor, in patients with mild to moderate knee osteoarthritis. The primary efficacy endpoints included the progression of joint space narrowing in the osteoarthritic knee, as measured by microfocal radiography with fluoroscopic positioning, and the reduction of symptoms (pain and stiffness) and/or the improvement of function, as measured by the Western Ontario and McMaster Universities osteoarthritis index (WOMAC). Four hundred and one patients were randomly assigned to either placebo (*n *= 80) or one of fourdoses of PG-116800: 25 mg (*n *= 81), 50 mg (*n *= 80), 100 mg (*n *= 80), or 200 mg (*n *= 80) taken twice daily for 12 months. During the study, the 200-mg dose was discontinued based on an increased frequency of musculoskeletal adverse effects. After 1 year of treatment, no statistically significant difference was observed between placebo and PG-116800 with regard to mean changes in minimum joint space width of the knee or to WOMAC scores. The most frequent adverse effect was arthralgia (35%). Twenty-three percent of evaluable patients had at least a 30% decrease from baseline of at least onerange-of-motion measurement of either shoulder at a follow-up visit. The percentage of patients with reduction in range of motion was significantly greater in the twohighest dose groups relative to placebo. Thirteen percent of patients, half of whom were in the 200-mg group, reported hand adverse events (oedema, palmar fibrosis, Dupuytren contracture, or persistent tendon thickness or nodules). The threemost frequent shoulder adverse events were reversible arthralgia, stiffness, and myalgia, which mostly affected the twohighest dose groups. The unfavorable risk-benefit balance of the MMP inhibitor PG-116800 in patients with knee osteoarthritis precludes further development of the compound for this indication. This study adds to the weight of evidence suggesting that side effect profiles of MMP inhibitors in general make them unsuitable for use in osteoarthritis.

ClinicalTrials.gov NCT00041756.

## Introduction

Osteoarthritis (OA) is a chronic, progressive disorder of the synovial joints, characterized by focal loss of cartilage and changes in subchondral and marginal bone, synovium, and periarticular structures [1]. The disorder commonly affects weight-bearing joints and results in pain and loss of function [2]. Current therapies (analgesics and nonsteroidal anti-inflammatory drugs [NSAIDs]) are mostly symptomatic and include adjuvant interventions such as weight loss and physical therapy to improve physical function. As the understanding of the pathogenesis of joint destruction in OA increases, new therapeutic approaches are targeting the tissue degradation process.

Matrix metalloproteinases (MMPs) are a group of approximately 30 proteolytic enzymes, which collectively degrade all the components of the extracellular matrix during tissue formation and remodelling [3,4]. As degradation of the extracellular matrix is essential for growth and progression of malignant tumors, MMP inhibitors have been extensively studied as potential anticancer agents [4]. MMPs have also long been implicated in the joint destruction process that occurs in arthritis, and MMP inhibitors have been studied in the treatment of both rheumatoid arthritis and OA [5-7]. Increased levels of MMP-1 and MMP-3 have been observed in the cartilage and synovium of patients with OA and have been correlated with the severity of the condition [8]. Animal models have shown that MMPs were good therapeutic targets [9]. Despite promising preclinical data [10-13], however, development of MMP inhibitors in arthritis seldom continued into humans, hampered by safety issues or an unfavorable efficacy profile in other indications [14]. To our knowledge, no controlled long-term studies with MMP inhibitors in OA have been performed to date.

In long-term trials in oncology, the use of MMP inhibitors has been associated with musculoskeletal toxicity, which was the primary reason for program termination for some compounds of this class [15,16]. Signs and symptoms described in the literature generally consisted of musculoskeletal pain and inflammation, usually originating in the upper shoulder girdle or hands, and also arthralgia, myalgia, joint stiffness, limb pain, Dupuytren contracture, and/or peripheral pain and oedema [4,17-23]. In published studies, musculoskeletal symptoms tended to occur after 2 to 3 months of treatment, to be dose-dependent, and to be generally reversible within 1 to 3 weeks of treatment discontinuation [4,17-19,23].

PG-116800 is a member of the hydroxyproline-based hydroxamic acid class of MMP inhibitors which has been shown to inhibit the joint damage caused by iodoacetate injection into rat knees, an experimental model for OA [24]. PG-116800 has high affinity for MMP-2, -3, -8, -9, -13, and -14, the therapeutic targets, while having substantially lower affinity for MMP-1 and -7, both of which were thought to be implicated in the pathogenesis of musculoskeletal toxicity [25].

This randomized, double-blind, placebo-controlled, multicenter, parallel-group, dose-response study was conducted to evaluate the relative efficacy of PG-116800 versus placebo in slowing the progression of joint space narrowing in knee OA (structural objective) and in reducing symptoms and/or improving function (symptomatic objective) over the course of 12 months and to assess the safety of its administration. The minimum joint space width (JSW) in the medial compartment of the tibiofemoral joint was measured from microfocal knee radiographs obtained by fluoroscopic positioning. Microfocal radiography was chosen for this study since investigators had shown that quantitative evaluation of measurements obtained from macroradiographs detected JSW changes within a shorter time frame than was possible using standard radiography [26,27]. Here, we report one of the largest studies of MMP inhibitors and describe the adverse musculoskeletal effects of the drug.

## Materials and methods

### Design and patients

Men and postmenopausal and/or hysterectomized women, 40 to 80 years of age, with primary OA of the knee according to the American College of Rheumatology criteria [28] were eligible to participate in the study. For the purpose of the radiographic assessment, patients had to have at least one knee that could be designated a 'signal knee' on microfocal x-ray, as defined by at least oneosteophyte in either the medial or the lateral compartment of the tibiofemoral joint (tibial spine osteophytes could be included), a JSW in the medial tibiofemoral compartment greater than or equal to 2 mm and less than 4.5 mm in the semiflexed view, and a medial compartment JSW that was narrower than the lateral compartment JSW for the same knee. Exclusion criteria included secondary OA, nonosteoarthritic causes of knee pain, significant medical or psychiatric conditions, chronic shoulder disease, severe obesity, previous intra-articular injection of either knee or of any other joint with steroids, previous intra-articular injection of the signal knee with hyaluronic acid, diagnostic arthroscopy, use of systemic steroids or knee injury within 3 months of study entry, history of surgery or surgical arthroscopy of the signal knee, and recent therapy with a drug of the tetracycline class, with calcitonin, systemic fluoride, bisphosphonate, glucosamine, digoxin, warfarin, inhibitors, or inducers of cytochrome P450 3A4, 1,25 (OH)_2 _D_3 _or with more than 400 IU/day of vitamin D_2 _or D_3_.

The study was conducted in 16 centers in the UK and 8 centers in Hungary between July 2002 and February 2004 in compliance with International Conference on Harmonization guidelines, the US Code of Federal Regulations, European Community guidelines, and the Declaration of Helsinki. Each study site's ethics committee approved the protocol, and all patients provided written informed consent. An Independent Data Monitoring Committee (IDMC) monitored unblinded data for safety.

### Treatments administered

Eligible patients were randomly assigned to receive orally twice daily for 1 year onecapsule containing either placebo or one of fourdoses of PG-116800 (25, 50, 100, or 200 mg). The PG-116800 capsules were supplied by Procter & Gamble Pharmaceuticals (Mason, OH, USA) as white opaque gelatin shells containing PG-530742 (the dihydrated sodium salt PG-116800), lactose monohydrate (National Formulary, NF), and magnesium stearate NF. Placebo capsules were identical in composition and appearance to the capsules containing active drug but did not contain drug substance. During the in-life portion of the study (November 2003), the IDMC recommended discontinuing the 200-mg dose based on an increased frequency of musculoskeletal adverse effects.

### Outcome and safety assessments

The progression of joint space narrowing in the osteoarthritic knee (structural primary endpoint) was evaluated by measuring the 1-year change from baseline in minimum JSW in the medial compartment of the tibiofemoral joint of the signal knee, using microfocal knee radiographs obtained in the semiflexed position. To ensure proper alignment of the tibial plateau and centering of the tibial spines relative to the femoral notch, fluoroscopic positioning was performed before the radiograph was acquired. Study radiographers in twocenters (one in Hungary and one in the UK) performing microfocal x-ray underwent specific training and testing before participating in the study. The reduction of symptoms and/or improvement of function of the knee (symptomatic primary endpoint) was measured by the Western Ontario and McMaster Universities osteoarthritis index (WOMAC) total score (version3.1) at 1 year [29]. Secondary endpoints included the consumption of analgesics and NSAIDs as pain medication for OA and the Patient Global Assessment ('Considering all the ways your OA affects you, how have you been in the last 48 hours?').

Tolerability and safety were evaluated at each visit through interviews by the site personnel. All treatment-emergent adverse events were recorded, and their severity (mild, moderate, or severe) and relationship to the study drug (doubtful, possible, or probable) were graded by the investigator. Clinical laboratory tests, electrocardiograms, and chest radiographs were performed regularly.

The occurrence of musculoskeletal effects was carefully monitored because of musculoskeletal toxicity reported with other MMP inhibitors in oncology trials [17]. Based on a literature review, a working definition of the MMP inhibitor-associated musculoskeletal syndrome (MSS) was developed and included painless loss of range of motion (ROM) in large joints (particularly in the shoulders), joint stiffness and joint swelling, soft tissue pain, and fibrosis of palmar tendons (Dupuytren contracture). To assess potential musculoskeletal toxicity, each follow-up visit included serial measurements of shoulder ROM (anterior flexion, abduction, internal rotation, and external rotation) at the site by an appropriately trained person using a goniometer; examination of palmar tendons for evidence of palmar tendon fibrosis, tendinitis, fasciitis, or inflammation; and elicitation of MSS symptoms through questioning. A diagnosis of MSS was considered likely if a patient (a) developed palmar fibrosis, (b) lost at least 30% of any shoulder ROM, as measured by goniometer, or (c) presented with musculoskeletal signs or symptoms the investigator considered to be significant and consistent with those previously reported in the literature. No specific blood biomarker of MMP inhibitor-related musculoskeletal toxicity was identified and collected. Patients with signs and symptoms consistent with MSS could undergo ultrasound of the hands and shoulders at the investigators' discretion and the sponsor's recommendation. The IDMC reviewed unblinded study data quarterly to enable the early discontinuation of doses that appeared to be associated with any unacceptable adverse events, with particular attention to any evidence of MSS.

### Sample size determination

The study was designed to detect a 50% reduction in JSW change at 1 year with 80% power, using one-sided comparisons, each made at an α value of 0.10 (not adjusted). The sample size of 75 patients per group (for a total of 375 enrolled) assumed a yearly loss in JSW of 0.2 mm, with a standard deviation of 0.25 mm. Treatment groups were sized to accommodate a 20% dropout rate. In addition, without regard for multiple outcomes, the study had 80% power to detect a 17% effect of treatment versus placebo with respect to pain modification, as measured by the pain subscale of the WOMAC index (assuming one-sided comparisons, each made at an α value of 0.10, a placebo mean of 160 mm, and a standard deviation of 70 mm).

### Randomization method

Patients were randomly assigned to fivetreatment groups using an Interactive Voice Response System (IVRS). Randomization of patients was balanced according to their current use of estrogens or selective estrogen-receptor modulators (SERMs) in which twostrata were formed. An adaptative randomization technique [30] was employed to better achieve treatment balance within each site, each estrogen/SERM stratum, and across the entire study. The pooled study center was treated as a stratification factor for all applicable efficacy endpoint analyses. The treatment codes were controlled by the clinical supplies department of Procter & Gamble Pharmaceuticals.

### Blinding

This was a double-blind study with limited access to the randomization code. Study drug was dispensed according to the instructions provided via the IVRS. The treatment each patient received was not disclosed to study personnel, participants, contractors (except for clinical supplies distributor and IVRS contractors), or the sponsor (except for select clinical supplies, pharmacovigilance, bioanalytical, and pharmacokinetics personnel).

### Statistical analysis

An intent-to-treat (ITT) analysis was conducted on all patients who were randomly assigned and took at least one dose of study drug. Any missing data for these patients were not imputed in the primary analyses. Thus, the efficacy analyses were based on the ITT patients with observed data at the time point under consideration. Paired *t *tests were used to test change from baseline values.

The 1-year change from baseline in minimum JSW in the medial compartment of the tibiofemoral joint of the signal knee was determined from the 12-month radiograph back to baseline. The significance of the 1-year change from baseline in minimum JSW and WOMAC total score was estimated using an analysis of variance. Minimum JSW change estimates were adjusted for baseline JSW, pooled center, and baseline use of estrogen or SERM drug replacement therapy as covariates. WOMAC change estimates were adjusted for baseline total scores, pooled center, and baseline use of estrogen or SERM drug replacement therapy as covariates. Each dose group was compared with the placebo control group. Each of these comparisons was made using a one-sided test with an α value of 0.10. Secondary endpoints were analyzed in a similar fashion.

Safety analyses included the ITT population. ROM measurements were analyzed and adjusted for pooled study centers as covariates. Comparisons between treatment groups were made to placebo for each visit. Additionally, the proportion of patients with detectable decreases in ROM was summarized. The proportion of patients with palmar tendon fibrosis, palmar tendon tendonitis, or palmar tendon fasciitis was summarized. Adverse events were tabulated and summarized according to the Medical Dictionary for Regulatory Activities (MedDRA). Percentages of patients reporting a decrease in shoulder ROM were compared to placebo by means of the Fisher exact test. Changes in joint symptom severity were tabulated and summarized, and percentages of patients reporting an increase in joint symptoms were compared to placebo by means of the Cochran-Mantel-Haenszel test after adjusting for pooled centers. *P *values for safety analyses were provided as flags for further investigation and were not adjusted for multiple testing.

## Results

A summary of patient accountability is presented in Table 1. Of 401 patients randomly assigned into the study, 395 took at least one dose of study drug (ITT population). The per-protocol population comprised 296 patients. Twenty-one patients in the 200-mg dose group were withdrawn from the study for safety reasons per IDMC recommendation, which was classified as a major protocol deviation. All patients were analyzed according to group assignment. Overall, treatment groups were balanced with regard to demographics and baseline characteristics (Table 2). The majority of patients were postmenopausal females who were not using hormone replacement therapy.

**Table 1:** Patient accountability

	Placebo	25 mg	50 mg	100 mg	200 mg	Overall
	n(%)	n (%)	n (%)	n (%)	n (%)	n(%)
Patients randomly assigned	80	81	80	80	80	401
Patients who took at least onedose of study drug	77	80	79	80	79	395
Patients in per-protocol population^a^	66	67	64	64	35	296
Patients completing 12 months of dosing	69 (90%)	65 (81%)	66 (84%)	64 (80%)	19 (24%)	283 (72%)
Patients who withdrew	8 (10%)	15 (19%)	13 (16%)	16 (20%)	60 (76%)	112 (28%)
Reason for withdrawal						
Adverse event	4 (5%)	10 (13%)	6 (8%)	11 (14%)	34 (43%)	65 (16%)
Protocol violation	1 (1%)	1 (1%)	2 (3%)	2 (3%)	1 (1%)	7 (2%)
Voluntary withdrawal	3 (4%)	3 (4%)	4 (5%)	2 (3%)	3 (4%)	15 (4%)
Lost to follow-up	0 (0%)	1 (1%)	0 (0%)	1 (1%)	0 (0%)	2 (<1%)
Unable to meet protocol criteria	0 (0%)	0 (0%)	1 (1%)	0 (0%)	1 (1%)	2 (<1%)
IDMC recommendation	0 (0%)	0 (0%)	0 (0%)	0 (0%)	21 (27%)	21 (5%)

n (%) = number and percentage of patients. % = n/N × 100.

^a^Per-protocol population refers to patients who completed the study as per protocol, including 75% compliance with randomized study drug during the course of the trial.

IDMC, Independent Data Monitoring Committee.

**Table 2:** Demographic and baseline characteristics (intent-to-treat population)

Parameter	Placebo	25 mg	50 mg	100 mg	200 mg
	(*n *= 77)	(*n *= 80)	(*n *= 79)	(*n *= 80)	(*n *= 79)
Age in years, mean (standard error of the mean)	62.0 (0.92)	62.4 (0.86)	62.6 (0.92)	62.9 (0.95)	63.1 (0.80)
Males, number (percentage)	23 (30%)	20 (25%)	18 (23%)	23 (29%)	30 (38%)
Females, number (percentage)	54 (70%)	60 (75%)	61 (77%)	57 (71%)	49 (62%)
Estrogen/SERM use among females, number (percentage)					
Yes	5 (9%)	9 (15%)	6 (10%)	5 (9%)	4 (8%)
No	49 (91%)	51 (85%)	55 (90%)	52 (91%)	45 (92%)

SERM, selective estrogen receptor modulator.

### Efficacy results

Overall, PG-116800 administration did not result in slower progression of joint space narrowing compared with that observed with placebo. There was no statistically significant difference between placebo and any of the PG-116800 groups in mean change in minimum JSW of the signal knee after 1 year of treatment (Table 3). The decrease from baseline in mean JSW of the signal knee was observed in all dose groups at month 12 and was statistically significant in all with the exception of the 25-mg group. Similarly, analysis of the symptomatic primary endpoint did not demonstrate statistically significant differences between placebo and each of the PG-116800 groups on total WOMAC scores (Table 4). Analysis of secondary endpoints showed that Patient Global Assessment scores improved significantly from baseline over the course of 12 months in all groups with the exception of the 200-mg group, but there were no differences between treatment groups (data not shown). At month 12, the consumption of pain medication for OA had decreased from baseline in all groups, the change from baseline reaching statistical significance in the placebo group only (data not shown).

**Table 3:** One-year change from baseline in joint space width of the signal knee (intent-to-treat population)

Treatment group	Number^a^	Joint space width at baseline	Joint space width at month 12	One-year change from baseline	Mean 1-year percentage change from baseline	*P *value^b ^(90% CI)
		Mean (SEM)	Mean (SEM)	Least square mean (SEM)		
Placebo	71	3.386 (0.085)	3.252 (0.096)	-0.136^c ^(0.063)	-4.00	
25 mg	66	3.350 (0.094)	3.307 (0.105)	-0.044 (0.062)	-1.24	0.127 (-0.012, ∞)
50 mg	67	3.346 (0.094)	3.123 (0.113)	-0.226^c ^(0.065)	-7.07	0.869 (-0.193, ∞)
100 mg	69	3.413 (0.094)	3.218 (0.129)	-0.200^c ^(0.065)	-7.61	0.789 (-0.166, ∞)
200 mg	52	3.466 (0.108)	3.278 (0.138)	-0.192^c ^(0.072)	-6.72	0.740 (-0.166, ∞)

^a^Number of patients with available data at baseline and month 12. ^b^One-sided *P *value: The mean change from baseline in each treatment group was compared to placebo by means of an analysis of variance after adjusting for pooled centers, baseline use of estrogen replacement therapy or selective estrogen receptor modulator drugs, and baseline joint space width. ^c^The mean change from baseline was significantly different from zero using a paired *t *test within each treatment group.

CI, confidence interval for mean difference; SEM, standard error of the mean.

**Table 4:** One-year change from baseline in total WOMAC scores (intent-to-treat population)

Treatment group	Number^a^	WOMAC total score at baseline	WOMAC total score at month 12	One-year change from baseline	*P *value^b ^(90% CI)
		Mean (SEM)	Mean (SEM)	Least square mean (SEM)	
Placebo	67	41.8 (2.16)	32.9 (2.78)	-9.1^c ^(2.81)	
25 mg	61	42.6 (2.90)	35.9 (3.21)	-6.7^c ^(2.78)	0.746 (-∞, 7.0)
50 mg	62	49.1 (2.46)	37.1 (3.20)	-10.0^c ^(2.88)	0.403 (-∞, 3.7)
100 mg	62	50.9 (2.66)	42.4 (3.10)	-5.8^c ^(2.92)	0.823 (-∞, 8.0)
200 mg	30	47.2 (3.70)	36.5 (4.54)	-9.3^c ^(3.96)	0.483 (-∞, 5.6)
25 mg versus placebo					0.485^d^
50 mg versus placebo					0.631^d^
100 mg versus placebo					0.374^d^
200 mg versus placebo					0.610^d^

^a^Number of patients with available data at baseline and month 12. ^b^One-sided *P *value: The mean change from baseline in each treatment group was compared to placebo by means of an analysis of variance after adjusting for pooled centers, baseline use of estrogen replacement therapy or selective estrogen receptor modulator drugs, and baseline total scores. ^c^The mean change from baseline was significantly different from zero using a paired *t *test within each treatment group. ^d^One-sided *P *value was based on between-group comparison of mean change from baseline by means of repeated-measures analysis adjusted for pooled centers, baseline total scores, and baseline use of estrogen replacement therapy or selective estrogen receptor modulator drugs.

CI, confidence interval for mean difference; SEM, standard error of the mean; WOMAC, Western Ontario and McMaster Universities osteoarthritis index.

### Safety results

The proportion of patients reporting adverse events was greatest in the twohighest dose groups (Table 5). Serious adverse events and withdrawals due to adverse events occurred with the highest frequency in the 200-mg group, in which adverse events were the primary cause of withdrawal. The majority of adverse events, serious adverse events, and withdrawals due to adverse events were musculoskeletal and connective tissue disorders. Arthralgia was the single most frequent adverse event overall (35% of all patients) and was also the most frequent serious adverse event and reason for withdrawal. Two patients, one in the 100-mg group and one in the 200-mg group, died of pulmonary embolism and cardiac failure, respectively, during the study. The investigators considered their deaths to be unrelated to study drug.

**Table 5:** Summary of adverse events (intent-to-treat population)

	Placebo	25 mg	50 mg	100 mg	200 mg
	(*n *= 77)	(*n *= 80)	(*n *= 79)	(*n *= 80)	(*n *= 79)
Patients with adverse events, number (percentage)	52 (68%)	51 (64%)	51 (65%)	62 (78%)	62 (78%)
Patients who withdrew, number (percentage)	8 (10%)	15 (19%)	13 (16%)	16 (20%)	60^a ^(76%)
Patients with serious adverse events, number (percentage)	8 (10%)	7 (9%)	12 (15%)	10 (13%)	17 (22%)
Patients who died, number (percentage)	0 (0%)	0 (0%)	0 (0%)	1 (1%)	1 (1%)

^a^Includes 21 patients withdrawn per Independent Data Monitoring Committee recommendation.

Objective monitoring of musculoskeletal toxicity included goniometer assessment of shoulder ROM at each visit. Of the 391 patients who had at least onevalid shoulder ROM measurement during a follow-up visit, 90 (23%) had a decrease of at least 30% from baseline for at least one ROM measurement of either shoulder (Table 6). The percentage of patients with changes in ROM was statistically significantly greater in the twohighest dose groups relative to placebo. Twenty-nine patients (7%) had an ROM decrease of at least 30% in both shoulders. Reproducibility of the decrease in shoulder ROM (that is, decrease of 30% in a measurement at twoconsecutive visits) was used as a practical measure of specificity of the finding. Of 376 patients with valid measurements, 40 patients (11%) had a decrease of at least 30% in the same measurement for either shoulder at twoor more consecutive visits. The decrease mostly affected shoulder internal and external rotations; fewer patients had decreases in shoulder abduction and anterior flexion. Finally, 34 of 391 patients (9%) had decreases of at least 30% in two or more ROM measurements of either shoulder at the same visit. The percentage of patients with two or more ROM decreases in the twohighest dose groups was statistically significantly greater relative to placebo (Table 6).

**Table 6:** Changes from baseline in shoulder range of motion (intent-to-treat population)

	Placebo	25 mg	50 mg	100 mg	200 mg
Patients with ≥30% decrease from baseline in ≥1 ROM measurement at any postbaseline visit for either shoulder, number (percentage)	12/77 (16%)	11/79 (14%)	18/78 (23%)	24/79 (30%)	25/78 (32%)
*P *value^a^		0.824	0.310	0.036	0.023
Patients with ≥30% decrease from baseline in the same ROM measurement at 2 or more consecutive postbaseline visits for either shoulder, number (percentage)	4/74 (5%)	5/78 (6%)	8/74 (11%)	12/77 (16%)	11/73 (15%)
*P *value^a^		1.000	0.367	0.062	0.061
Patients with ≥30% decrease from baseline in 2 or more ROM measurements at the same postbaseline visit for either shoulder, number (percentage)	2/77 (3%)	2/79 (3%)	5/78 (6%)	13/79 (16%)	12/78 (15%)
*P *value^a^		1.000	0.442	0.005	0.009

^a^Treatment groups were compared to placebo by means of the Fisher exact test. ROM, range of motion.

Adverse events affecting the hand were reported in 52 of 395 patients (13%), nearly half of whom were in the 200-mg group (Table 7). Common signs included hand oedema (5 patients), palmar fibrosis (15 patients), Dupuytren contracture (5 patients), and tendon thickening/nodules (3 patients). Most patients with hand involvement (16 patients) had asymptomatic nodules without function loss (contracture), hand pain, or hand oedema. Of the 7 symptomatic patients, 4 were in the 200-mg group, 2 in the 100-mg group, and 1 in the 50-mg group. A mild function loss in hands (mild contracture) was reported in 5 symptomatic patients, 2 of whom had accompanying pain and localized oedema, which were not reported as separate adverse events. Two other symptomatic patients included 1 patient with mild hand pain (localized hypersensitivity) and another patient with pain and oedema in the hand without any functional loss. The first cases of palmar fibrosis appeared at month3, and the number of affected patients in the 200-mg dose group reached statistical significance versus placebo at month9 (18% versus 1%; *p *= 0.001). The finding of fibrosis on hand palpation was confirmed by hand ultrasound in 20 patients. Ultrasound was not a scheduled procedure in the study protocol. The usual appearance of fibrosis on ultrasound consisted of elongated or oblique bands along flexor tendons, in their proximity but not within the tendons. Their size varied depending on the examination technique (palpation versus ultrasound) and ranged from a few millimeters to approximately 2 cm in length. In one case, a thickening of fascia palmaris of 6 cm in length was described.

**Table 7:** Patients with hand adverse events (intent-to-treat population)

	Placebo	25 mg	50 mg	100 mg	200 mg
Patients with hand adverse events, number (percentage)	5/77 (6%)	8/80 (10%)	9/79 (11%)	7/80 (9%)	23/79 (29%)
Patients with particular hand findings (hand oedema overlapped with other findings), number					
Hand oedema	0	0	1	1	3
Palmar fibrosis	0	1	1	2	12
Dupuytren contracture	0	1	0	3	1
Tendon thickness/nodules	0	0	0	0	2
Symptomatic patients, number	0	0	1	2	4

The threemost frequent shoulder adverse events were arthralgia, stiffness, and myalgia. The first cases of shoulder arthralgia, which affected either shoulder with similar frequency, appeared after 1 month of dosing. Over the course of the whole study, 13%, 16%, 13%, 21%, and 25% of patients, respectively, in the placebo, 25-mg, 50-mg, 100-mg, and 200-mg dose groups reported shoulder arthralgia. Shoulder stiffness, which was usually isolated but sometimes accompanied by stiffness in other joints, was reported starting at month6. Over the course of the study, 1%, 4%, 6%, 9%, and 6% of patients, respectively, in the placebo, 25-mg, 50-mg, 100-mg, and 200-mg groups reported shoulder stiffness. Shoulder myalgia generally affected the deltoid muscle and was reported in 1%, 1%, 4%, 3%, and 6% of patients, respectively, in the placebo, 25-mg, 50-mg, 100-mg, and 200-mg groups over the course of the study. The threemost frequently reported shoulder adverse events could represent different reporting of a similar symptomatology. An analysis combining the threemost frequent shoulder adverse events showed a dose-related response starting after 3 months of dosing, continuing through months6 and 9, and disappearing at month 12 (data not shown).

Other symptoms included neck pain (3%, 4%, 4%, 6%, and 10% of patients, respectively, in the placebo, 25-mg, 50-mg, 100-mg, and 200-mg groups), which could be due to referred pain originating in the shoulder, and shoulder periarthritis (1%, 3%, 1%, 4%, and 6% of patients, respectively, in the placebo, 25-mg, 50-mg, 100-mg, and 200-mg groups). Shoulder ultrasounds were performed at the investigator's discretion in some symptomatic patients. Of 13 symptomatic patients in whom rotator cuff rupture (partial or complete tear) was found on shoulder ultrasound during the study, 3 patients each were in the placebo, 50-mg, 100-mg, and 200-mg groups and 1 patient was in the 25-mg group. Few patients (7%) had a combination of shoulder and hand symptoms that appeared independent of each other.

Most patients withdrawn from the study because of development of musculoskeletal adverse events were followed until resolution or stabilization of symptoms. Cessation of MMP inhibitor administration seemed to bring symptomatic relief as well as improvement in ROM in patients with shoulder involvement. Among 19 patients followed up for clinically significant shoulder involvement, the improvement was complete in 10 patients after a mean period of 122 days and partial in 3 patients after a mean period of 107 days (partial means that the symptoms or signs decreased but never returned to baseline status) (Table 8). Additionally, 3 patients with shoulder involvement recovered completely while still exposed to active drug and 3 other patients recovered partially. Complete recovery was seemingly promoted by local glucocorticoid injections in 4 patients and physiotherapy in 5 patients. Partial recovery was promoted by local glucocorticoid injections in 1 patient and physiotherapy in another patient. Patients were also administered NSAIDs and paracetamol for the shoulder symptoms or index knee OA. Recovery from shoulder symptoms was faster in patients who were exposed to the MMP inhibitors for a shorter period of time. Six patients with shoulder involvement underwent temporary drug interruption followed by drug rechallenge. The symptoms recurred in 4 of these patients. Of the 21 patients followed for hand adverse events, only 2 recovered completely after a mean period of 224 days from their last dose of study drug. Eleven patients recovered partially after a mean period of 210 days and the hand fibrosis was unchanged in 7 patients after a mean period of 196 days after stopping the study drug (partial recovery means that either the symptoms decreased or the extent of fibrosis decreased in size in clinical or ultrasound assessment) (Table 8). Of the 3 patients followed for involvement of both shoulders and hands, 2 patients recovered partially after a mean period of 170 days from the last dose of study drug (partial recovery means that symptoms or signs in at least one site of involvement, usually the shoulder, decreased). Additionally, 1 patient with both shoulder and hand involvement recovered partially while still taking active drug. Initial symptoms of hand pain or oedema, which accompanied the diagnosis of hand fibrosis, tended to disappear after stopping study drug.

**Table 8:** Follow-up data after PG-116800 withdrawal in patients with clinically significant shoulder findings and hand adverse events

	Level of recovery	Number of patients	Mean time (days)	Median time (days)	Minimum time (days)	Maximum time (days)	Comments
Shoulder	Complete	10	122	81	5	316	Additional 3 patients recovered while still on drug
	Partial	3	107	138	30	154	Additional 3 patients recovered while still on drug
	Unchanged vs. baseline	0					
Hand	Complete	2	224	224	163	285	
	Partial	12	210	213	93	329	
	Unchanged vs. baseline	7	196	184	77	303	
Both	Complete	0					
	Partial	2	170	170	79	261	Additional 1 patient recovered while still on drug
	Unchanged vs. baseline	0					

## Discussion

This proof-of-concept and dose-ranging study failed to demonstrate efficacy of PG-116800, an MMP inhibitor, in modifying the course of knee OA in patients with mild to moderate disease during a 12-month treatment period, as determined by microfocal x-ray assessment of JSW and analysis of WOMAC total scores, despite promising results of nonclinical studies [9,10,12].

The study confirmed that microfocal radiography is a sensitive tool in detecting a decrease in JSW in osteoarthritic patients over the course of a 12-month period. The progression of OA as evidenced by a decreasing JSW coexisted with symptomatic improvement from baseline in all treatment groups, including placebo. Significant symptomatic placebo response has been observed in OA studies of both intra-articular and oral medications and was noted in other recent studies [31-34]. This study seems to confirm that patients are likely to improve symptomatically and that the placebo effect persists for at least 12 months. Progression of JSW narrowing accompanied by symptomatic improvement over the course of a 1-year time period calls for further research in surrogate markers of OA activity and progression.

Although the majority of the cases described in the literature have been associated with the use of marimastat in patients with cancer, musculoskeletal toxicity has been reported in association with most, if not all, other MMP inhibitors. Musculoskeletal effects have been observed in preclinical studies with other MMP inhibitors [35]. In preclinical studies conducted with PG-116800, swelling was observed around the joints of both rats and dogs during chronic toxicology studies (3 and 6 months in rats and 12 months in dogs;, Procter & Gamble Pharmaceuticals, unpublished data). In apparent relation to the swelling, rats and dogs had accumulation of collagen associated with the joint structures. In dogs, after 12 months of study, proliferation of periosteal fibrous tissue and resorption of bone were observed in the joint. The effects appeared to be reversible when observed at the end of a 3-month recovery period.

Because the potential musculoskeletal toxicity of MMP inhibitors was recognized before the study start, safety measures included shoulder ROM assessment and monitoring with an IDMC. Although a detailed hand examination was part of the baseline physical examination, investigators were sensitized to hand findings after cases of asymptomatic nodules were observed. IDMC-driven withdrawal of patients in the 200-mg dose group could result in cases of musculoskeletal toxicity being over-reported at withdrawal in this group following the unblinding. The hand findings had a striking clinical resemblance to those of early development of Dupuytren contracture.

One of the major challenges in identifying patients with drug-related musculoskeletal toxicity in the study population was the need to differentiate from the symptoms and signs of OA itself. Although upper girdle involvement in OA is less frequent than involvement of hips and knees, it is not uncommon for the two to coexist in the same patient. Additionally, in published series, up to 50% of asymptomatic patients more than 60 years of age had partial or full rotator cuff tears in shoulder imaging studies [36]. Since shoulder ultrasound was not part of the screening process in this study but was used for further workup in some patients reporting shoulder symptoms, the finding of a rotator cuff tear was not helpful in differentiating drug toxicity from age-related tissue degeneration.

The occurrence of the most frequently reported shoulder adverse events (arthralgia, myalgia, and joint stiffness) followed a time- and dose-response pattern. This pattern disappeared after 12 months. Early termination of the study by patients in the highest dose group as per the IDMC recommendation after most of the patients had been dosed for 6 months offers a plausible explanation together with early withdrawals of symptomatic patients.

A number of theories to explain the occurrence of the musculoskeletal effects observed with MMP inhibitors have been offered, but none of them has definitive proof to support it. Inhibition of sheddase activity attributed to hydroxamate-based MMP inhibitors might be responsible for musculoskeletal symptoms [4]. Sheddase activity converts membrane-bound cytokines and receptors to inactive forms. Therefore, inhibiting this conversion could result in inflammation. In support of this theory, patients treated with BMS-275291 (which has reduced activity against sheddases) initially did not experience MSS [4]. This finding was verified after longer treatment duration when similar adverse events were reported. Another theory was that musculoskeletal toxicity might be caused by MMP-1 inhibition [4]. In an attempt to minimize these effects, Bayer Corp. (West Haven, CT, USA) and Agouron Pharmaceuticals Inc. (La Jolla, CA, USA) developed tanomastat and prinomastat, which strongly inhibit MMP-2 and -9 and weakly inhibit MMP-1, -7, and -11. However, prinomastat continued to exhibit musculoskeletal toxicity, whereas tanomastat was abandoned for lack of efficacy in oncology indications [4,37].

## Conclusion

The study did not demonstrate efficacy of PG-116800, an MMP inhibitor, in the treatment of patients with knee OA. It indicated that musculoskeletal side effects compromise the safety of long-term (greater than 3 months) systemic administration of the compound. Shoulders were affected clinically, with a decrease in ROM and an increase in pain largely reversible upon drug discontinuation. A dose- and time-related focal accumulation of tissue consistent with palmar tendon fibrosis was observed after 2 to 3 months of treatment. These musculoskeletal side effects were similar to those reported for other MMP inhibitors. This is likely to reflect a class effect, as these adverse effects are now reported for most of the MMP inhibitors, and this further suggests that these agents are unlikely to be of practical clinical use for the treatment of OA.

## Abbreviations

IDMC = Independent Data Monitoring Committee; ITT = intent-to-treat; IVRS = Interactive Voice Response System; JSW = joint space width; MedDRA = Medical Dictionary for Regulatory Activities; MMP = matrix metalloproteinase; MSS = musculoskeletal syndrome; NF = National Formulary; NSAID = nonsteroidal anti-inflammatory drug; OA = osteoarthritis; ROM = range of motion; SERM = selective estrogen-receptor modulator; WOMAC = Western Ontario and McMaster Universities osteoarthritis index.

## Competing interests

PK, GAC, KS, RL, JB, and WSA were all employees of Procter & Gamble Pharmaceuticals when the study was conducted. TDS and CB-W were consultants to Procter & Gamble Pharmaceuticals at the time of this study. GB declares that he has no competing interests.

## Authors' contributions

GB and TDS were investigators in the study. CB-W developed the microfocal x-ray technique and reviewed all films. PK and JB were the medical monitors. KS and RL were the project leaders, participating in the study design and coordination. GAC performed the statistical analysis. WSA was medical advisor. All authors were involved in data interpretation and read and approved the final version of the manuscript.
